# Pregnancy and Labor Following Complete Nephro-Ureterectomy

**Published:** 1903-03

**Authors:** 


### Pregnancy and Labor Following Complete Nephro-Ureterectomy.

BOVEE (*Virginia Medical Semimonthly*, December, 1902), relates the case of a woman upon whom he operated March 18, 1901, for pyonephrosis, renal calculi and miliary abscesses in the ureter. She became pregnant the July following and was delivered April 15, 1902, of a male child weighing 10½ pounds, after a labor of six hours' duration. The third day after delivery she complained of intense pain along the course of remaining (left) ureter. This was accompanied by fever ranging from 100° to 102°. The fever declined to normal in about a week, the tenderness over the ureter remaining some time. There were no further complications and the patient was able to nurse her infant, and at the time of the report remained in splendid health. The urine during pregnancy showed albumen and a specimen examined three months later showed it still present, but no casts and with a normal percentage of solid.

